# Exploring Foot Care Conditions for People Experiencing Homelessness: A Community Participatory Approach

**DOI:** 10.1177/21501319211065247

**Published:** 2022-01-28

**Authors:** Melba Sheila D’Souza, Joyce O’Mahony, Alfred Achoba

**Affiliations:** 1 Thompson Rivers University, Kamloops, BC, Canada; 2 Executive Director, Canadian Mental Health Association, Kamloops

**Keywords:** access to care, community health, emergency visits, health outcomes, primary care, underserved communities

## Abstract

**Introduction:**

People experiencing homelessness are faced with complex challenges and are at high risk of illness due to inequities and disparities in access to health care services.

**Objective:**

To explore the health and foot care problems related to people experiencing homelessness in British Columbia.

**Methods:**

A community participatory research approach was used with a sample of 65 people experiencing homelessness. Data were collected using a survey questionnaire and face-to-face semistructured interviews.

**Results:**

Thematic findings shows risk of foot injuries, lack of foot care resources, and absence of family support. Barriers to equitable access to services for most participants experiencing homelessness were lack of housing (76.92%), inability to work (72.31%), and inability to afford the cost of living on their own (63.08%).

**Conclusions:**

There is a pressing need for early screening and detection by health care professionals and enhanced foot care services to reduce foot problems and improve foot care wellness of homeless people. Addressing foot-related care are necessary steps in promoting health, preventing illness, and improving access to health services among people experiencing homelessness.

## Introduction

Homelessness is caused by lack of affordable housing, unemployment, poor mental health, and low social support.^1^ Lack of access to necessities like food, clothing, shelter, transport, social services, and health care affect people’s lives. Hence, poverty, unemployment, and lack of housing and income is a vicious cycle of poverty affecting homeless people’s lives. Socioeconomic status is a significant contributing factor to mental health and health status. People experiencing homelessness have higher prevalence of physical health problems, including respiratory illnesses, musculoskeletal disorders, chronic pain, malnutrition, and infectious disease. Chronic illness such as diabetes, hypertension, poor dental hygiene, skin, and foot conditions are reported as higher compared to the general population.^2^ They are vulnerable to mental health changes, substance use, trauma, and domestic violence.^3^ Many people experiencing homelessness resort to remedial spaces such as foster care, community placements, and institutionalized care without adequate plans for a future safe dwelling to meet their needs. While health care services address several health care concerns of people experiencing homelessness, there is a lack of services that focus on this population’s foot-related health care needs.^4^

In 2018, 25 216 Canadians experienced absolute homelessness in shelters and 6789 people in transitional housing.^5^ People experiencing chronic homelessness accounted for 60% of all respondents, whereas episodic homelessness accounted for 8% of all respondents.^6^ One-quarter of respondents (25%) had not used a shelter in the past year. It is reported that 53% of people were less likely to stay in a shelter if they were staying with others. Many homeless choose not to access health care for foot care and health problems such as calluses, blisters, and trench foot.^7^ Commonly, health illnesses in homeless people have comorbidities such as respiratory conditions, skin problems, foot disorders, hypertension, cirrhosis, HIV, and injuries.^8^ People experiencing homelessness are exposed to social stigmatization, social isolation, unstable living conditions, chronic health problems,^9^ and difficulties accessing healthcare.^10,11^ Many homeless people are compounded by these complex challenges and at high risk of illness due to inequities and disparities in access to health services.^12^ Overall health is affected alongside the inability to receive foot care due to constant walking in poor weather conditions. In many instances it remains undetected thereby increasing foot related problems in people experiencing homelessness and ultimately affecting their quality of life. Conditions of shoes, socks, and foot odor was a significant deterrent to using foot care services.

Chen et al^13^ conducted a review of the literature and found 3 articles found poor foot conditions and foot infections among the homeless. Another study reported that 18 out of 21 people experiencing homelessness reported foot pain and foot ulcers.^14^ Six healthcare professionals were interviewed concerning foot care access experiences on the street and validated foot conditions were common.^15^ The barriers identified by 26 homeless men and women in the interview were limited income, limited health care access, lack of healthy food availability, lack of access to primary care physicians, and waiting on entitlement decisions.^16^ The prevalence of foot-related problems across people experiencing homelessness is corn and calluses (7.7%-57%), nail pathologies (15%-65%), infections (3.2%-38%), and injuries (24%-43%).^17^ People experiencing homelessness (9%-65%) are more likely to have foot infections, pain, functional limitations, bunions, hammertoes, gout, plantar warts, foot ulcers, and frostbite.^17^ They are at high-risk for developing foot problems, which could lead to further health-related consequences such as multimorbidity, surgery, amputation, increased hospitalization, and disability.^18^ People experiencing homelessness report feeling stigmatized and discriminated against by some health care professionals, which in turn prevents them from accessing health care supports and services.^19,20^ People experiencing homelessness with mental health illness and substance abuse feel further difficulties in accessing and finding trusting health care professionals.^21^ Thus people experiencing homelessness have poor access to health care, leading to higher utilization of emergency services.

Primary care is considered important for access and care of people who are homeless. It is viewed as an easier approach to health care services to improve health. There are currently few foot care services existing for people experiencing homelessness in the interior region of British Columbia. There are gaps in the literature regarding the social barriers to homeless people’s foot care needs and stakeholders’ perspectives, and the provision of homeless people services. Community and health services often overlook foot care needs of people experiencing homelessness, which leads to increased utilization of emergency services and deterioration in their health. Since people experiencing homelessness are underrepresented in health service research, little is known about their unmet health care needs, particularly in smaller cities where resources are limited. The objective of this study is to explore barriers and facilitators of foot care conditions related to people experiencing homelessness in British Columbia.

## Methods

### Ethical Considerations

This research study was reviewed and approved by the institutional research ethics board. The participants were free to withdraw their participation at any time and without any further consequences to present or future health care services. All participants received an information letter describing the study and participant expectations, informed consent, and the opportunity to ask questions at any time. Confidentiality and privacy were maintained and safeguarded throughout the study. This was done by assigning code-names during data transcription to remove identifiers and prevent linkages to protect participants.

### Setting and Design

A community participatory approach^22^ was used to explore the social determinants of health and health care access among homeless people in British Columbia. This community-oriented approach included collaboration with community organizations, homeless participants, and academic partners. All collaborators worked and played a role in community engagement with homeless people. This approach was advantageous for recruitment of participants, campaigning, and networking with interested community healthcare professionals. The community stakeholders offered various community supports such as housing, shelters, medical aid, social work, outreach center, living accommodation, rehabilitation, a kitchen, and a health center initiative for people experiencing homelessness.

### Sampling

A purposive sample was used to recruit homeless people from shelters and housing and drop-in centers through the managers of the organizations. A total sample size of 65 was obtained. Eligible homeless study participants were defined as adults who were above 18 years, sheltered in transitional houses, spoke English, were able to provide written informed consent, and could participate voluntarily. Vulnerable people experiencing domestic violence or abuse or had active mental health problems or cognitive impairment were excluded from the study.

### Data Collection Tools

By consulting the literature, community providers, and social workers, a healthcare semi-structured interview guide was developed which included items on health care access, foot experiences, self-care, coping, family support, and psychosocial support. Socio-economic and community services survey was developed and validated with a community health nurse and an outreach nurse. The community services survey was found to be reliable with *r* = .78 using an inter-rater reliability coefficient.

### Data Collection

Data were collected using a community services survey and a healthcare semi-structured interview. Data collection took place between September 2019 and February 2020 in the interior of British Columbia. Informed written consent was obtained before engagement in the study. The principal investigator spent 2 days a week for 6 months visiting the community organizations. Privacy, dignity, and comfort were ensured in a private space during the interviews and surveys.

### Data Analysis

All interviews were transcribed verbatim. To analyze the data, qualitative analysis method^23^ was used to look for patterns to explain phenomena under investigation. Open coding generated the selection and named categories to yield datasets. First, interview transcripts were read and re-read to build familiarity with the data. Second, establishing a coding template by using consistencies and divergences between diverse participant perspectives. This assisted generating codes and outlining patterns and categories to compare within and across all transcripts. Third, iteratively identifying and refining themes and generated broader theoretical reformulations for the emerging results. This involved interpreting the findings, looking for patterns, finalizing the themes, and supporting the findings’ credibility, dependability, and trustworthiness. This final step acted as a type of member-checking stage to increase data validity and trustworthiness. The interview data were transcribed and entered NVivo 12 (qualitative data analysis software) by the research assistant and double-checked by the primary investigator.

## Results

### Socio-Economic and Community Services Survey

#### Socio-economic data

Based on the demographic data, 30.8% of the people experiencing homelessness were female, 60% had completed middle school, and 38.5% reported a history of foot problems and emergency visits (Table 1). People experiencing homelessness had moved to and were living in the small city due to the availability of social services (53.85%), having been born and raised in the city (47.69), work opportunities (29.23%), and having family or friends (23.08%). They reported familiar places for staying at night between April and October were shelters (78.46%), someone else’s home (36.92%), on the street (26.15%). Some indicated that they became homeless after being discharged from Armed Forces, rehabilitation centers, recovery homes, hospitals, jails, or prisons (15.38%) (Table 2). People experiencing homelessness also reported their current living situation included sheltered (81.54%), streets (29.23%), and home of a family member or friend (21.54%) (Table 2).

**Table 1.** Demographic Characteristics and Living in Cities Among Homeless People N = 65.

Demographic	Variable categories	Frequency (Percentage)
Age	1: ≤50 years	26 (40%)
	2: >50 years	39 (60%)
Years of homelessness	1: ≤5 years	26 (40%)
	2: >5 years	39 (60%)
Gender	1: Male	45 (69.2%)
	2: Female	20 (30.8%)
Years of schooling	1: Grade 8: Middle School	39 (60%)
	2: Up to Grade 11: High School	13 (20%)
	3: Grade 12: Higher Secondary, Vocational/Diploma	13 (20%)
History of foot problems and emergency visits	1: Yes, foot problems and visits	25 (38.5%)
	2: No foot problems and visits	40 (61.5%)
Reasons for living in smaller cities	Frequency	Percentage
Access to social services	35	53.85
Born and raised in smaller cities	31	47.69
Work opportunities and conditions	19	29.23
Family/friends live here	15	23.08
I knew someone who lives here	12	18.46
Education/training	5	7.69
Access to health care services	4	6.15
Others	4	6.15

**Table 2.** Stay at Nights and Living Situations Among Homeless People N = 65.

Stay at nights (April-October)	Frequency	Percentage
Shelter, safe home, transition home	51	78.46
Someone else’s place, relative, family, friends, couch surfing	24	36.92
Streets/Outside/Open spaces	17	26.15
Makeshift shelter, a tent in open spaces parks, riverside	6	9.23
In a vehicle (Car, Van, Camper, Recreational vehicle)	2	3.08
Adult foster home	2	3.08
Parents/guardians home if the youth is under 25 years	4	6.15
Discharged from Armed forces, Detox, recovery home, hospital, jail, institution	10	15.38
Post-traumatic stress disorder (PTSD)	1	1.54
Natural disaster	1	1.54
Current living situation	Frequency	Percentage
Your vehicle/camper vehicle	11	16.92
Shelter	53	81.54
Home of a relative/friend	14	21.54
Streets	19	29.23
Adult foster care	1	1.54
Other	12	18.46

#### Community services data

The barriers to equitable access to services for most of the people experiencing homelessness were lack of housing (76.92%), inability to work (72.31%), and inability to afford the cost of living on their own (63.08%) (Table 3). People experiencing homelessness indicated several barriers to accessing foot care services. These included not having a steady income (49.23%), major life-changing event (eg, kicked out of house, theft, incarceration) (38.46%), loss of job (36.92%), active mental health concerns (29.23%), or could not find a job (24.62%). The enablers to accessing equitable services were having affordable or safe housing (84.62%), community navigators or resources (63.08%), treatment for addictions (40%), medical coverage (33.85%), immediate health care (32.31%), and helpline information (27.69%) (Table 4). Other enablers to participation and utilization of equity-oriented services included having food services (64.62%), housing services (55.38%), mental health services (52.31%), general health services (46.15%), employment and financial services (35.58%), and drop-in centers (23.08%) (Table 4).

**Table 3.** Access to Social and Health Care Services Among Homeless People N = 65.

Barriers to equity access/services	Frequency	Percentage
Loss of job	24	36.92
No income/funding/poverty	32	49.23
Evicted from the rental home	14	21.54
Not able to work	47	72.31
No work available	16	24.62
Significant life-changing event (eg, divorce, theft, incarceration) Something happened in life	25	38.46
Cannot afford costs on their own	41	63.08
No housing	50	76.92
No food security	13	20
No childcare	5	7.69
Intimate partner violence	2	3.08
Active mental health problems	19	29.23
Family well-being	5	7.69

**Table 4.** Enablers to Social and Health Care Services Among Homeless People N = 65.

Enablers to equity access	Frequency	Percentage
Affordable/safe housing	55	84.62
Training and education	5	7.69
Steady employment	10	15.38
Addictions and treatment	26	40
Medical coverage	22	33.85
Good roommate	1	1.54
Peer support groups	3	4.62
Community navigators/resources	41	63.08
Local help desk/helpline information	18	27.69
Access to urgent/immediate health care	21	32.31
Access to urgent/immediate foot care clinics	5	7.69
Utilization of equity services	Frequency	Percentage
Health services (Ambulance, Emergency room, Hospital (non-emergency), Dental)	30	46.15
Mental health (Addiction, Health clinic)	34	52.31
Food services (Food banks, Meal programs/soup kitchens)	42	64.62
Legal services (Legal, Parole or ex-offenders)	1	1.54
Employment and financial services (Employment/job help, Budgeting/trusteeship)	23	35.38
Housing services (Housing help/eviction prevention, Homeless outreach, Transitional housing)	36	55.38
Other services (Drop-in, Newcomer services, Faith-based/spiritual services)	15	23.08

#### Healthcare semi-structured interview

Three themes emerged from the data analysis, including increased risk of foot injuries, lack of foot care resources, and absence of family support.

##### Theme 1: Risk of foot injuries

People experiencing homelessness expressed they visited the emergency room when they were injured, or during episodes of illness. These people had a range of foot problems related to skin, circulation, sensation, nails, diabetes, pain, blisters, burning, redness, and sores attributed to walking many miles.

Participants revealed various issues:

*“I had addiction in the past, and I got stuck on it. I could not work and one day someone ran a motorbike over my right foot. It still hurts me, and I think the bone may be broken. I ended up in the emergency room. I am not a bad person, I had to go through bad situations. I just need my pain medications. Once I had a bad foot injury and was in the hospital and had to rest my foot. I feel that I need to look after my feet, but I have no control over my foot problems. I cannot stop drinking; I drink a lot. I drank too much on one occasion and fell off the curb into the gulley. I woke up in the emergency room. I did not feel comfortable with the way they interviewed me regarding my drinking and drug issues. I feel anxious in this situation”.* PH21

Another participant experiencing homelessness stated,

*“My foot was injured, years back, when I was playing football two years back. It developed an infection over the years. Years later, I had a fall, and my left foot was injured at the construction site. They drove me to the emergency room. They said that I had blood clots and was treated with medications. It has been a long time to find out what has been happening to my foot and leg. I have been waiting for a long time for some scans and surgery. They know that I am unwell”.* PH34

Challenges of walking to different locations and having problems sleeping were challenges voiced by another participant

*“I have to walk for my daily needs, but the cold or heat is exhausting and tiring. I had broken by back at my previous workplace. So walking is painful to me and getting anywhere is difficult with my back and knee pain. I have had bad feet with swelling and pain. It is sometimes embarrassing to ask for pain medications, I do not like to ask for it. I try not to see the doctor. I have to search for a place to find the medications that I need.”* PH07

##### Theme 2: Lack of foot care resources

One participant described that their feet were soaking wet and had worn out socks in the cold and snow: “I could not take off my shoes on the ground. Someone will steal them and had to sleep with feet inside the shoes.” Another person echoed the same response: “I never remove my shoes off my feet.” Some people experiencing homelessness expressed burning their feet and toes while sleeping near an open campfire, embarrassed about having street feet or trench feet, frostbite, and a foul smell of feet. These types of foot conditions can prevent people experiencing homelessness from seeking appropriate health care. Several participants related their experience to common experiences shared

*“My feet are immersing in snow and soggy. I catch my feet soaking in wet socks in the cold. I cannot take off my shoes; Someone will steal them. I sleep in with my shoes on all day and night. I burnt my toes twice when I slept near a campfire. I had callouses, red patches and blisters on my feet due to the cold weather. If the person who is homeless are not in the shelters or on the streets where the street nurses visit, they miss getting treated by the street nurses and then must go to the walk-in clinics or emergency room. The walk-in clinics open very early and have a short appointment list. If they [SU] are late, you cannot see the doctor”.* PH39

Another people experiencing homelessness recalled:

*“I get cold, frozen feet, and chills in the winter. I have had frostbite and frozen toes in the cold. My feet are wet and soggy in the cold. This is the new normal for me. I do not ask for help, because I do not need anyone. Some days I go with out eating much when I cannot panhandle. I am careful to use the public showers with my recurrent foot fungus, which gives off a bad smell. I walk all day that causes street feet, and I rub some oil into it or soak it in Epsom salt when I find some. I do not have a place to wash my socks or clothes. I wear a used pair of socks until they are completely worn out. I have bad feet, and my shoes are shabby and not in good shape. My foot problems grow daily. I need a warm place to care for myself”.* PH1

Similarly, another participant described:

*“The *foot sores and burning were always present. I stay in my shoes all day, which makes it itchy, and I cannot take my shoes off for fear of losing them. I did not have a place to wash my feet and hands. There is no washroom for me or to wash my feet. I make friends with other homeless to survive in the cold. I stay on the streets in the day and under the bridge at night. I beg for money from kind people every day at the crosswalk signals. I do not feel safe to keep my shoes and socks here. I must be friendly with people. There are no washrooms and no way to shower or clean clothes or socks. I am looking for a place to take off my shoes and socks to let my feet air* dry”.* PH07

##### Theme 3

Absence of family support.

People experiencing homelessness who were not sheltered talked about hardship, deprivation, and poverty. They talked about their struggles and challenges living on the street and being stigmatized.

One participant talked about local resources and stated,

*“There are local resources around here for homeless people like me. They offer services and support to homeless people. I try to connect with different services for my needs. I like living in this small city. Its easy to find your way in the crowd downtown. I lost my identification card, and they helped me get another one. I had posttraumatic stress disorder and medications. I had been in bad situations before. I talk to homeless people about where to look for food or help. If I get any part-time job, that will help me get my own place, something that I had not had in a long time.”* PH34

Another participant shared how difficult it is to keep clean and stay on necessary medications:

*“There are no washrooms for us and no way to clean or shower or change. I have not been to a mental health doctor for years and homeless. I was on medications for my mental health illness. Once I was discharged, there was no way to continue the medications. I was on and off medications, and I missed my appointments. I did not return to my doctor. I learnt how to do things my way. I am not sensitive and not weak. I know how to get around the system to do some extent of good to myself. I can survive.”* PH41

Another participant hints at being stigmatized yet at the same time knows where to get food and resources when needed:

*“I had been depressed and shy. I can not help myself. Sometimes I get very depressed. I stay under the bridge. I have not seen my doctor for my diabetes. Sometimes I panhandle at the crossroads in the city, and I go to places when they offer food. I clean my foot by the riverside at the park downtown. On winter days I struggle to find a dry place.”* PH19

There were multiple quotes regarding missing family members, lack of friends, feelings of depression, child visitation restraints and overwhelming emotional and spiritual issues. One participant hoped to settle in a smaller city and was looking for a job with social services supports. However, his fear, intimidation, and psychological problems were barriers to obtaining employment. Obtaining adequate sleep and rest was a challenge and impacted day to day living and ways of coping:

*“I spend time by myself. I stay far from trouble. I get along well with some homeless people. Sometimes I worry too much. I have a hard time sleeping. I do not get attention from my family. I feel lonely and not having any friends. I feel depressed and low, thinking that I am away from home. I do not feel energetic and feel drained out.”* PH57

Another participant lamented about her disconnection with family members

*“I did not get along well with my dad and my mom died. I lost my husband seven years back. I lost it with my kids, and the social services took them away. I yelled at my kids and scared the hell out of them. Now I cannot see my kids for a long time. I am homeless. I have a sister that lives in the mainland. She does not talk to me”.* PH12

While another people experiencing homelessness expressed that,

*“It is difficult not having a roof over my head, to feel safe and protected. I lost my job and I have been on the streets since then. I do not know any kinds of job. All my life, I only knew to take care of my kids and cook. My kids live in another province.”* PH38

Some people experiencing homelessness reported feeling isolated and alienated from other homeless people. One participant mentioned that:

*“I am not a people person; I cannot please people. I say outright what I think and feel. I do not like being around people. I spend time by myself. If I talk to others, they try to get close to me and take my belongings. Sometimes they harass you and steal my things. It is not safe living on the streets. I do not talk to others, and I stay away from them.”* PH21

Another person shared:

*“I do not talk to other homeless people, and I do not get close to them. They sometimes stir trouble. Some homeless people have mental health issues because they have no one to care for them. Some of them do not know the system that they live in. They think its a lot of waiting time to hear about their social disability monies”.* PH23

## Discussion

In this study, self-awareness of poor foot hygiene contributes to the stigma of being homeless and their shared feelings of shame and defeat. This stigma results in a lack of motivation by participants to reach out to whatever community resources available. People experiencing homelessness attributed a lack of access to health care providers with not having health insurance, lengthy wait for an appointment, and lack of a disability status. Further contributors to poor foot care were lack of family and social support due to irregular income, unemployment, and diminished motivation to participate in self-care activities. Mental health illness further exacerbated the situation for some people experiencing homelessness. Prevention of foot problems with limited resources were a challenge for many participants in this study. Access to daily hygiene was unreliable, and therefore a high risk of tinea pedis and scabies as shoes and socks were worn day and night. They did not feel safe to remove their shoes and socks during the night. Foot hygiene was poor with limited resources and home-made remedies such as vinegar, garlic, or olive oil. Some people experiencing homelessness air-dried their feet and tried to remove shoes and socks when they found a safe place city park open spaces or secluded beaches. In other studies, homeless people did not have access to a primary care provider.^24^ Younger homeless people accessed the emergency room for medical conditions.^25^ Increased likelihoods to use emergency rooms was seen more with increased age, chronic years of homelessness, and recurrent history of foot problems.^26^

In this study, people experiencing homelessness often faced multiple barriers simultaneously such as lack of housing, inability to work/job loss, unaffordable living costs, inconsistent income, adverse changes in their life, no safety net, and exacerbating mental health concerns. People experiencing homelessness had limited access to health care with infrequent follow-ups, missed medical appointments, expensive medication prescriptions. They faced challenges related to maintaining personal hygiene, lack of cleanliness, and grooming. In other studies, fragmentation, lack of identifying documents,^27^ stigma and discrimination,^28^ ethnic and racial identity disparities^29^ contribute to long wait times, and reduced access create additional barriers for these vulnerable people.^30^

Enablers to support homeless people were access to affordable and safe housing, inclusion of community navigators and peer support, treatment for substance use and mental health, increased medical and health coverage, and access to immediate care and information. Some people experiencing homelessness had limited education and were not aware of how to access health care and social services. They may have had a negative experience with healthcare services in the past. Other studies showed higher number of people experiencing homeless, including 60.8% of men and 96.2% of women living in shelters, whereby 28% to 34% were Indigenous, and 4.3% of the Canadians are Indigenous.^31^ Forty-nine percentage of people living in shelters were adults, and 13% were dependent and unaccompanied youth.^32^ One study showed a direct effect of education and healthcare access on self-perceived health status.^26,33^

Most people experiencing homelessness reported that they did not own a house to live in, could not work, and could not afford costs on their own. Nearly half of the participants did not have a steady income, lost their job, active mental health concerns, and/or could not find suitable employment. Previous studies report low income/education, food insecurity, unsafe housing, and social exclusion influences access to equity-oriented services. Other studies demonstrated that homelessness had been linked to unstable employment and lack of affordable health care.^1^ Alcohol and drug abuse, mental and physical illness, and neglect, deprived backgrounds,^34^ crimes,^35,36^ can lead to homelessness.^1^ In other studies, physical ability, perceived importance, patient knowledge, education provision, social integration, risk status, and patient-provider communication were identified as enablers of foot self-care.^18^ Previous studies show that unemployment, food and shelter insecurities, mental illness, and lack of social and family support limit their capacity to deal with demanding aspects of management in low-income, vulnerable, marginalized, and high-risk populations.^37^ Poverty, lack of social and community support,^34,35,38^ physical and emotional abuse, neglect, dysfunctional families, and unstable family structures increased the likelihood of homelessness.^39^ Other studies showed a lack of access to health services^28^ and financial resources homeless people from receiving appropriate treatment; 74% of homeless people walked an average of 5 hours each day.^13^

Researchers had challenges with timing of the recruitment of people who are homeless living in the interior region. This was due to . . . Results of this study cannot be generalized to all people experiencing homelessness or to other contexts due to the geographical setting. Despite these limitations, the results of this study contribute significantly to raise awareness and understanding of what would be helpful in meeting the foot care needs of people experiencing homelessness.

### Implications for Practice

A community and primary care partnership framework are recommended to explain the social determinants of health and bridge the challenges of self-managing and residing in the community for people experiencing homelessness. There is an urgent need for early screening and detection by health care professionals and foot care services for homeless people in order reduce foot problems and improve foot care wellness. Addressing foot-related care are necessary steps in promoting health, preventing illness, and improving access to health services among homeless people. It is essential to assess for foot-related issues upon examination that may reveal foot and skin problems that can be readily treated and improve quality of life. Clean foot accessories, hygiene, and appropriate referral services would be part of the shelters’ foot care outreach services. The provision of supportive housing and community resources will help to alleviate the foot care problems and enhance quality of life of people experiencing homelessness. A community based participatory research approach is needed to further assess feasibility and effectiveness with specific foot care clinics for people experiencing homelessness in smaller cities. Evaluation of existing social and health care environmental resources for people who are homeless can be explored. Future recommendations would be to assess the sustainability of mobile foot care clinics for marginalized sub-populations in remote, rural, and sub-urban areas.

## Conclusions

This study contributes to insights from people who experience with lived experience of homelessness and their foot care needs and challenges. The themes derived from the study show challenges with obtaining basic physiological needs such as foot problems, and bodily pain and discomfort. Understanding the social determinants of health can positively enhance interdisciplinary professionals’ attitudes for a humanistic and empowering approach for people experiencing homelessness. Various social determinants of health also impact homeless people, such as limited income, low social status, poor health knowledge, lack of housing, decreased social support and coping skills, unhealthy behaviors, and inequitable access to health services. The study showed multifactorial triggers of foot problems for people experiencing homelessness. Immersion foot, frostbite and “street feet,” job insecurity, food insecurity, inadequate housing, and low work supports were insufficient to satisfy their priority needs. The study has identified challenges to foot care in homeless people that could be helpful for planning better practices, strategies, and policies to improve foot care access. Our findings highlight critical elements for homeless people with unmet health needs, including health equity, coordination, and relationships within the health and social context. Findings show that navigation through complex healthcare presented a substantial barrier when people who are experiencing homelessness are unaware of how-to self-advocate for the foot care they require.
